# Third *Streptococcus pneumoniae* disease outbreak involving serotype 4–ST801 in a shipyard, Finland, May to June 2025

**DOI:** 10.2807/1560-7917.ES.2025.30.41.2500758

**Published:** 2025-10-16

**Authors:** Maria Francesca Manca, Jaakko Silvola, Jakub Czarnecki, Joana Sequeira Neto, Mari Kanerva, Heikki Kaukavuori, Ana Cristina González Pérez, Irmeli Lindström, Heikki Frilander, Mikhail Fomichev, Harri Marttila, Riitta Ratia, Leif Lakoma, Lotta Siira

**Affiliations:** 1 ECDC Fellowship Programme, Field Epidemiology path (EPIET), European Centre for Disease Prevention and Control (ECDC), Stockholm, Sweden; 2 Finnish Institute for Health and Welfare (THL), Helsinki, Finland; 3 Department of Clinical Microbiology, Turku University Hospital, The Wellbeing Services County of Southwest Finland (Varha), Turku, Finland; 4 ECDC Fellowship Programme, Public Health Microbiology path (EUPHEM), European Centre for Disease Prevention and Control (ECDC), Stockholm, Sweden; 5 Department of Infectious Diseases and Infection Control, Turku University Hospital, the Wellbeing Services County of Southwest Finland (Varha), Turku, Finland; 6 Meyer Turku, Turku, Finland; 7 Finnish Institute of Occupational Health (FIOH), Helsinki, Finland; 8 Clinical Chemistry, Emergency and Automation Laboratory, The Wellbeing Services County of Southwest Finland (Varha), Turku, Finland

**Keywords:** *Streptococcus pneumoniae*, invasive pneumococcal disease, occupational outbreak, shipyard, case-control, whole genome sequencing, vaccination

## Abstract

Finland experienced three invasive pneumococcal disease (IPD) outbreaks among shipyard workers at the same shipyard, in 2019, 2023 and 2025. During the latest outbreak (30 April–6 June 2025), 13 cases were reported, with six confirmed. All five isolates from blood culture-positive cases were serotype 4 – sequence type 801. These were nearly indistinguishable from three isolates from the 2019 outbreak, nine Finnish IPD surveillance isolates and isolates of a 2019 Norwegian shipyard outbreak. We found an association with welding.

From 30 April to 6 June 2025, 13 cases of pneumococcal pneumonia among workers in a shipyard in Turku were reported to the Finnish Institute for Health and Welfare (THL). In 2019 and 2023, two pneumococcal disease outbreaks in the same shipyard were investigated, involving 37 cases implicating serotypes 4, 12F and 8, and 14 cases implicating serotypes 4 and 9V, respectively [1,2]. We describe the recent 2025 outbreak and explore the genomic similarity between the outbreaks and invasive pneumococcal disease (IPD) surveillance isolates.

## Outbreak detection and public health measures

On 9 May 2025, the Wellbeing Services County of South-west Finland (Varha) notified a suspected outbreak of five cases of pneumococcal pneumonia among workers in Turku shipyard, starting 3 May 2025. In this area, 24 IPD cases are notified on average every year in the working age population (20–64-years-old). At the time, almost 9,000 workers representing 95 different nationalities could access the shipyard. The workers were employed either by the shipyard or by one of more than 1,200 subcontractors.

### Case definition

A probable case was defined as an individual with a radiologically confirmed lower tract infection compatible with signs of pneumococcal pneumonia, such as lobar pneumonia, diagnosed since 15 April 2025 and up to 2 months after the last case was diagnosed, and who had worked in the Turku shipyard since 1 April. A confirmed case had, in addition, microbiological confirmation through culture or nucleic acid amplification detection of *Streptococcus pneumoniae* from blood and/or a positive pneumococcal urinary antigen test.

### Clinical characteristics

**Figure 1:**
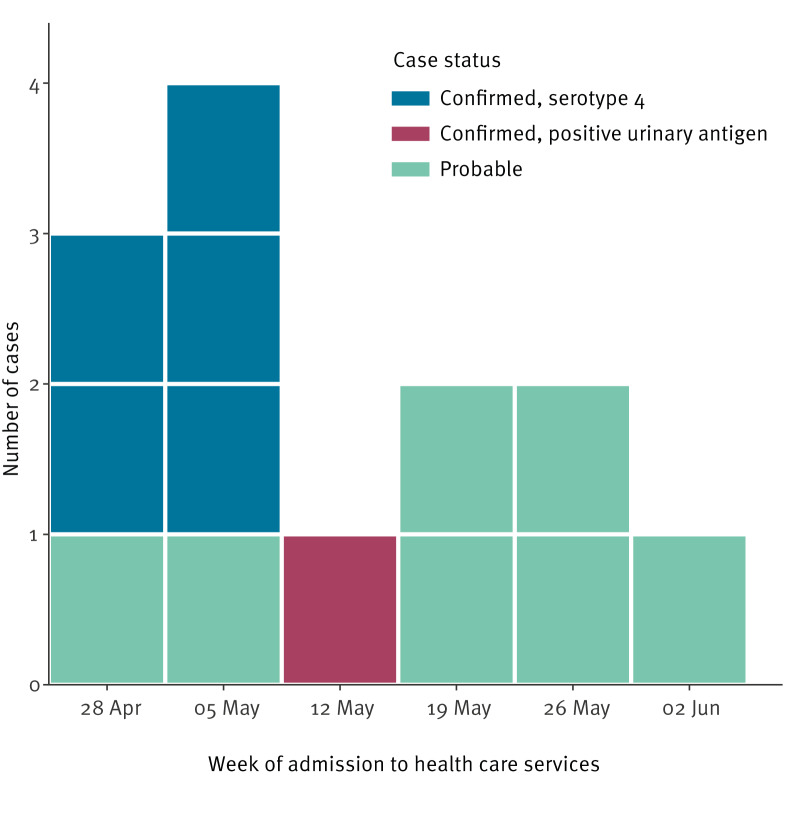
Pneumococcal pneumonia cases at a shipyard, by week of admission into healthcare services, Turku, Finland, 3 May–6 June 2025 (n = 13)

We identified six confirmed and seven probable cases (Figure 1). Eleven cases were admitted to care at Turku University Hospital (TYKS), three of them in the intensive care unit. The remaining two cases presented to primary healthcare.

Blood cultures from 12 cases were analysed at the TYKS clinical microbiology laboratory. Five were positive for *S. pneumoniae*. One case had a positive pneumococcal urinary antigen test. Two co-infections with respiratory syncytial virus, one with influenza B, and one with rhinovirus were reported.

All cases were male, their age ranged from 21 to 59 years (median: 41 years). Six cases were nationals of Ukraine, two of Finland and the remaining five of Bulgaria, Belarus, Latvia, the Philippines and Thailand.

### Vaccination campaign

A vaccination campaign using the pneumococcal conjugate vaccine 20 started on 26 May 2025, targeting up to 4,000 shipyard workers. On 6 June 2025, when the campaign ended, 1,005 individuals (25%) had been vaccinated. In addition, occupational healthcare vaccinated around 250 workers.

### Risk communication

Varha issued a press release on 23 May 2025, and THL notified of the outbreak through EpiPulse and the Early Warning and Response System on 30 May 2025.

## Epidemiological investigations

We conducted a case–control study in the shipyard during the vaccination campaign. Controls were shipyard workers recruited at the vaccination site and in one canteen, on a voluntary basis. No further selection criteria were applied for recruitment, and we did not set a predetermined number of controls to minimise the risk of missing data. Self-administered online or paper questionnaires were available in Finnish, English, Russian and Polish with supporting staff available on site speaking nine different languages. A copy of the questionnaire is appended in the Supplement.

Nine cases were interviewed by Varha using the second questionnaire appended in the Supplement. For the remaining four cases, only limited information about their profession, sector of work, smoking and vaccination history was available.

We described key characteristics of cases and controls and performed univariable and multivariable analyses to identify risk factors by calculating crude odds ratios (cORs) and adjusted ORs (aORs). Demographic variables and variables with p < 0.10 in univariable analysis were entered into the multivariable logistic regression model. Values of p < 0.05 were considered significant (Table).

**Table:** Factors associated with being a case, *Streptococcus pneumoniae *outbreak in Turku shipyard, Turku, Finland, 3 May–6 June 2025

		Exposed among cases (n = 13)		Exposed among controls (n = 192)		Univariable model			Multivariable model		
Characteristic		n	%	n	%	cOR	95% CI	p value	aOR	95% CI	p value
Age (years)		43 (IQR: 40–58)		39 (IQR: 30–50)		1.04	1.00–1.10	0.078	1.08	0.97–1.23	0.2
Sex											
		n = 13		n = 183							
Female		0	0	25	14	Reference			Reference		
Male		13	100	158	86	9,515,407	0.00–Inf	> 0.9	12,586,083	0.00–Inf	> 0.9
Nationality											
		n = 13		n = 192							
Finland		2	15	112	58	Reference			Reference		
Other		11	85	80	42	7.70	2.00–50.6	0.009	2.97	0.26–46.0	0.4
Living situation											
		n = 9		n = 191							
Alone		3	33	38	20	1.22	0.26–4.20	0.8	Excluded from the final model		
With other persons		6	67	153	80	0.22	0.07–0.69	0.009	0.93	0.05–28.4	> 0.9
Employer											
		n = 12		n = 190							
Shipyard		1	8	61	32	Reference			Excluded from the final model		
Contractor		11	92	129	68	5.20	0.98–96.2	0.12	Excluded from the final model		
Tasks at workplace											
Welder		n = 9		n = 186							
		4	44	34	18	3.58	0.85–14.2	0.068	23.8	1.76–1,026	0.038
Plumber		n = 11		n = 186							
		7	64	21	12	13.7	3.83–56.3	< 0.001	2.39	0.21–24.3	0.4
Welding ≥ 1–2 h/day		n = 9		n = 176							
		7	78	55	31	7.70	1.79–52.8	0.013	1.09	0.06–23.6	> 0.9
Sector of work											
		n = 9		n = 184							
Outfitting in tents		1	11	28	15	0.70	0.04–4.01	0.7	Excluded from the final model		
Outfitting in halls		2	22	31	17	1.41	0.20–6.17	0.7	Excluded from the final model		
Hull production		1	11	28	15	0.73	0.04–4.22	0.8	Excluded from the final model		
Wet dock		4	44	99	54	0.69	0.17–2.67	0.6	Excluded from the final model		
	Machinery	1	11	23	13	0.88	0.05–5.09	> 0.9	Excluded from the final model		
	Interior decks	3	33	27	15	2.91	0.59–11.7	0.15	Excluded from the final model		
		n = 11		n = 184							
Dry dock		7	64	74	40	2.60	0.76–10.2	0.14	Excluded from the final model		
		n = 9		n = 184							
	Machinery	4	44	16	9	8.40	1.92–35.0	0.003	4.67	0.15–119	0.3
	Interior decks	2	22	21	11	2.22	0.32–9.92	0.3	Excluded from the final model		
Other workplace-related factors											
Time spent at the shipyard ≤ 1 year		n = 8		n = 192							
		3	38	46	24	1.90	0.38–8.06	0.4	4.15	0.25–85.1	0.3
Borrowing personal protective equipment from colleagues		n = 9		n = 182							
		1	11	19	10	1.07	0.06–6.32	> 0.9	Excluded from the final model		
Lunch at the workplace		n = 8		n = 181							
		4	50	143	62	0.27	0.06–1.17	0.070	0.14	0.01–1.75	0.2
Other risk factors											
Alcohol consumption		n = 9		n = 186							
		7	78	162	87	0.52	0.12–3.62	0.4	Excluded from the final model		
Former or current smoker		n = 11		n = 181							
		5	46	75	41	1.18	0.33–4.05	0.8	Excluded from the final model		
Underlying comorbidities		n = 9		n = 166							
		4	44	30	18	3.63	0.85–14.5	0.066	5.04	0.31–101	0.2
Commute to work											
		n = 8		n = 181							
By bike or walking		1	13	19	10	1.22	0.06–7.39	0.9	Excluded from the final model		
By car alone		2	25	92	51	0.32	0.05–1.44	0.2	Excluded from the final model		
By car with colleagues		4	50	48	27	2.77	0.63–12.1	0.2	Excluded from the final model		
By public transport		1	13	22	12	1.03	0.05–6.20	> 0.9	Excluded from the final model		
Socialising with colleagues outside of work											
		n = 9		n = 184							
Daily or almost daily		1	11	19	10	1.09	0.06–6.40	> 0.9	Excluded from the final model		
2–3 times a week		0	0	10	5	0.00		> 0.9	Excluded from the final model		
2–4 times a month		4	44	23	13	5.60	1.31–22.7	0.015	2.87	0.10–64.2	0.5
≤ once a month		1	11	68	37	0.21	0.01–1.20	0.15	Excluded from the final model		
Never		3	33	64	35	0.94	0.19–3.68	> 0.9	Excluded from the final model		
Vaccination											
		n = 8		n = 184							
During 2019 campaign		0	0	38	21	0.00	NA	> 0.9	Excluded from the final model		
During 2023 campaign		1	13	19	10	1.24	0.06–7.52	0.8	Excluded from the final model		
At the shipyard, outside of campaigns		0	0	4	2	0.00	NA	>0.9	Excluded from the final model		

aOR: adjusted odds ratio; CI: confidence interval; cOR: crude odds ratio; NA: not applicable.

Cases and controls were comparable according to most questionnaire variables. However, Finnish nationals were significantly more common among controls and plumbing tasks were significantly more frequent among cases.

Based on univariable analysis, working as a plumber, welding at least 1–2 h every day, working on machinery and technical decks in the dry dock, socialising with colleagues outside working hours 2–4 times per month were significantly associated with being a case. Living with other persons was negatively associated with being a case. In multivariable analysis, only performing welding tasks was significantly associated with being a case.

## Microbiological investigations

The five blood culture isolates were sent to the expert laboratory at THL, where they were serotyped by the *Quellung* method (SSI Diagnostica, Denmark). Whole genome sequencing was done on the MiSeq (Illumina, United States) platform. The serotypes were predicted using the PneumoCaT algorithm and multilocus sequence typing (MLST) was done using stringMLST [3]. Core genome MLST (cgMLST) using the SeqSphere+ v.10.0.5 software (Ridom GmbH, Germany) was visualised in a minimum spanning tree [4].

**Figure 2:**
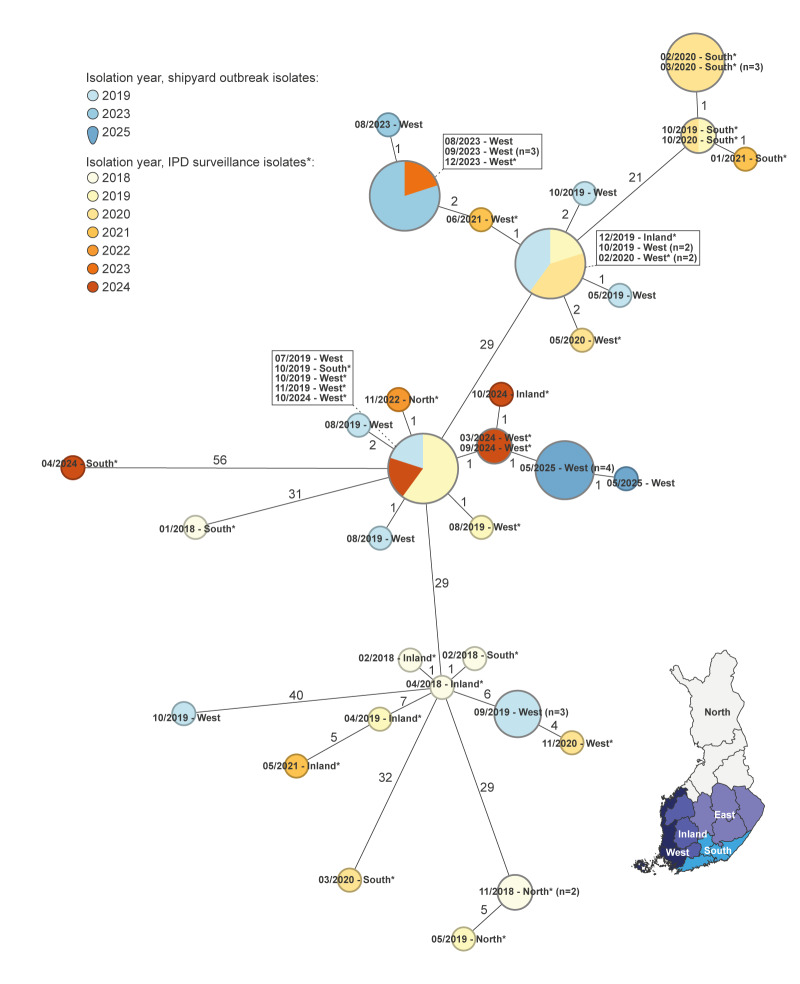
Minimum spanning tree of serotype 4–ST801 *Streptococcus pneumoniae* isolates from shipyard outbreaks in 2019, 2023 and 2025, and invasive pneumococcal disease surveillance isolates from 2018 to 2024, Finland (n = 55)

All isolates were vaccine serotype 4 – sequence type (ST) 801. Isolates from 2025 (n = 5) were compared with serotype 4–ST801 isolates from the outbreaks in 2019 (n = 11) and 2023 (n = 5), and with serotype 4–ST801 IPD surveillance isolates between 2018 and 2024 (n = 34) from Finland. All isolates of the 2025 outbreak were closely related with 0–1 allelic differences (AD) (Figure 2). They showed high similarity (< 5 AD) with three isolates from the 2019 outbreak and with nine surveillance isolates from sporadic cases during the period 2019 to 2024.

IPD: invasive pneumococcal disease.

The analysis is based on core genome multilocus sequence typing analysis including 1,079 targets. Isolates are colour-coded by combined date of detection and origin (surveillance or outbreak) of respective cases. The nodes size corresponds to the number of isolates. Isolates are identified by month and year of collection, and collaborative area (North, East, Inland, West and South) as defined by the application instructions on cooperation agreements between wellbeing services counties (https://stm.fi/en/collaborative-areas). Surveillance isolates are tagged with an asterisk(*).

## Further developments

On 12 September 2025, 1 month after the end of the follow-up period, Varha reported two new cases of pneumococcal pneumonia among shipyard workers, one of them serotype 4.

## Discussion

The epidemiological curve of the 2025 outbreak shows a peak of confirmed cases during the first days, unlike in the previous outbreaks [1,2].

A case–control study conducted during the 2023 outbreak identified as risk factors living in an apartment/studio or hotel/hostel rather than in a house or with family, and having worked at the shipyard for less than 1 year [2]. In 2025, we identified task–related risk factors, such as plumbing and welding. However, the small number of cases and potential selection bias of controls – as we recruited most of them at the vaccination site, despite additional recruitment at a canteen – restrict the strength of our conclusions. For four cases, we only disposed of limited information as they were not available for interviews. Our study is also limited by the imprecision of radiological findings to diagnose pneumococcal pneumonia, in absence of positive blood culture, nucleic acid detection or positive urinary antigen. Nevertheless, the risk factors we found have also been described in the literature: pneumococcal disease outbreaks have been associated with working outside, welding and exposure to welding dust and fumes [5-10].

Notably, *S. pneumoniae* serotype 4–ST801, has been involved in different pneumococcal outbreaks in shipyards [1,2,11,12]. The isolates from the Finnish 2019 outbreak which are very similar to those from the current outbreak, were previously reported as nearly indistinguishable from 13 isolates from a 2019 pneumococcal disease outbreak in a shipyard in Norway [11]. Concerning surveillance data, increased incidence of serotype 4 has been observed globally in all adult age groups, and an increase in ST801 was observed in the United Kingdom (UK) between 2017 and 2023 [13,14].

In all three Finnish outbreaks, vaccination campaigns were rapidly organised [1,2]. Vaccination uptake has decreased from over 4,000 workers in 2019 and over 3,000 in 2023, to 1,500 in 2025. However, workers vaccinated in 2023 were not eligible in 2025, which partly explains this decrease.

Besides vaccination campaigns implemented during outbreaks, a recommendation to vaccinate all new shipyard workers has been in place since 2019 [2,15]. In other countries, such as the UK, Germany and Norway, pneumococcal vaccination is recommended for welders [16,17].

According to the Finnish Occupational Safety and Health Act, the employer is responsible for ensuring workplace safety, including identifying and preventing or mitigating biological risks [18]. Based on risk assessment, the employer is obligated to arrange and fund vaccinations to protect an employee from substantial health risks. Despite this legislation and the abovementioned recommendation to offer pneumococcal vaccines to new shipyard workers, the understanding of workplace exposures as a risk factor for severe disease and outbreaks has been insufficient, as this and our previous studies show that vaccines are not routinely offered to new shipyard workers [1,2]. Discussions and exploration are underway to extend the occupational healthcare card in use in the construction sector in Finland to the shipbuilding industry, as is the full revision of the communicable diseases law. This could offer a way to increase the oversight of vaccination of shipyard workers.

## Conclusions

Our findings confirm a persistent undetected circulation of pneumococcal serotype 4–ST801 in the shipyard, where it repeatedly causes outbreaks. Our study also suggests a broader presence in the community in Finland. Recurrent pneumococcal outbreaks in shipyards underscore the need to implement public health measures, especially routine immunisation, among those at occupational risk of severe pneumococcal disease. Moreover, the occurrence of two new pneumococcal disease cases shortly after this outbreak, shows the need of continuously monitoring the situation by building awareness among potentially concerned workers and physicians, pursuing case interviews and rapidly typing isolates.

## Data Availability

The sequencing data for this study are available in the European Nucleotide Archive (ENA) at EMBL-EBI under accession numbers, PRJEB43223, PRJEB35348 and PRJEB76834.

## Supplementary Data

471000 Supplement
