# Supplementary material for: Third Streptococcus pneumoniae disease outbreak involving serotype 4–ST801 in a shipyard, Finland, May to June 2025
Source: Euro Surveill. 2025 Oct 16;30(41):2500758. doi: 10.2807/1560-7917.ES.2025.30.41.2500758 (PMC12534778; doi:10.2807/1560-7917.ES.2025.30.41.2500758)
Supplement: Supplement [file 25-00758_MANCA_Supplement.pdf]

SUPPLEMENTARY MATERIAL

This supplementary material is hosted by Eurosurveillance as supporting information alongside the article “Third Streptococcus pneumoniae invasive disease outbreak involving serotype 4 sequence type 801 in a shipyard in Finland, May-June 2025”, on behalf of the authors, who remain responsible for the accuracy and appropriateness of the content. The same standards for ethics, copyright, attributions and permissions as for the article apply. Supplements are not edited by Eurosurveillance and the journal is not responsible for the maintenance of any links or email addresses provided therein.

SUPPLEMENT S1: QUESTIONNAIRE FOR CASES pages 2-8

SUPPLEMENT S2: QUESTIONNAIRE FOR CONTROLS pages 9-15

S1: Questionnaire for pneumococcal disease outbreak at the Turku Shipyard in Turku, Finland, May-June 2025

Date of completion: (dd/mm/year): _____/_______/________
Case ID: ________
Interviewer: ________________________________

Good day, my name is [**name of interviewer**]. I am calling on behalf of [**name of institute**]. Is this Mr/Ms **[name of case]**? I am calling regarding the pneumonia you have recently been treated for in Turku University Hospital.

We are investigating the recent situation at the shipyard because we received reports that you were not the only person that got sick.

We would appreciate your input by answering a questionnaire. Answering the questions is voluntary, your participation will be useful to understand why the disease was identified among shipyard workers. Would you help us by participating?

(Yes)
Just for your information, any details you provide will be handled completely confidentially and will not be shared with your employer or any other party. Any information about you will have a number on it instead of your name. In reports of the findings, no individual participants will be identifiable. Any questions regarding the investigation can be addressed to [**the interviewer**] or to the THL/Varha investigation team. By answering the questions, you confirm that consent to participate is given.

(Yes, but not right now)
I will call you back, what time and day would you prefer?

(No)
Just for your information, any details you provide the investigation team will be handled completely confidentially and will not be shared with your employer or any other party. Any information about you will have a number on it instead of your name. In reports of the findings, no individual participants will be identifiable. In case you change your mind regarding your participation, you can contact this number [**xxxxxxxxxxx**].

Section A: Respondent information

1. Date of birth (dd/mm/year): ___ ___ / ___ ___ / ___ ___ ___ ___
2. Sex: __male / female / other / do not want to disclose___________________
3. Nationality: _____________________
4. Town or municipality of residence in Finland: _____________________
5. What date did the respiratory symptoms start? (dd/mm/year): _____________________
6. When did you start working at Turku Shipyard? (month, year) _____________________

The below information concerns the period of 3 months before start of symptoms (questions 6-7):

7. Where do you live when you work?
   - ☐ Hotel/hostel
   - ☐ Apartment/studio
   - ☐ House
   - ☐ Other: ________________

8. When you work, do you live:
   - ☐ Alone
   - ☐ With work colleagues (how many: ____)
   - ☐ With family members (how many: ____)
   - ☐ With other roommates that do not work at Turku Shipyard (how many: ____)

Section B: Occupational details / work-related questions

9. What is your employer?
   - ☐ Meyer Turku
   - ☐ Contractor (if a contractor, please specify **name** of the contractor and which **country** the contractor is based in): ____

10. Did you work in any other shipyard within the last 12 months?
    - ☐ Yes (if yes, please specify where and when): ____
    - ☐ No

11. Since you have started working in the shipyard, have you had occupational health checks?
    - ☐ Yes
    - ☐ No

12. Can you describe your main tasks/work in the shipyard?

    The below information concerns the period of 3 months before start of symptoms:

    Please mark all performed activities during this period:
    - ☐ hitsaaja -welder
    - ☐ levyseppä –plater
    - ☐ operaattori (automatisoidun hitsaus/polttoleikkauskoneen käyttö)- operator (in automated use of welding/oxy-fuel cutting machine)
    - ☐ putkiasentaja- plumber/pipefitter
    - ☐ koneasentaja- mechanic
    - ☐ sähköasentaja- electrician
    - ☐ lvi-asentaja- HVAC-technician/mechanic
    - ☐ sisustusasentaja – interior technician/mechanic
    - ☐ maalari ja ruiskumaalari –(industrial) painters or spray-painters
    - ☐ kunnossapito (sähkö-, nosturi-, koneasentajia)- maintenance and repair (electricians, crane-mechanics, mechanics)
    - ☐ varastomiehiä- storage workers
    - ☐ kuljetusmiehiä (trukki, nosturi)- transportation (crane and forklift-drivers)
    - ☐ siivooja – cleaner

☐ hiekkapuhallus - sandblasting
☐ Any other activities:
____

13. Which sector(s) of the shipyard do you work in?
The below information concerns the period of 3 months before start of symptoms:

☐ **Outfitting ship on wet dock (Varustelutyö laiturissa olevassa laivassa)**
- ☐ Machinery & Technical decks (konevarustelu)
- ☐ Interior & Passenger decks (varustelutyö, sisustus ja hyttikannet)
- ☐ Electrical outfitting (sähkövarustelu)
- ☐ HVAC & catering, kitchen areas (varustelutyö, LVI ja keittiöalueet)
- ☐ Deck outfitting (kansivarustelu)

☐ **Outfitting ship on dry dock (Varustelutyö allasvaiheen laivassa)**
- ☐ Machinery & Technical decks (konevarustelu)
- ☐ Interior & Passenger decks (varustelu sisustus ja hyttikannet)
- ☐ Electricial outfitting (sähkövarustelu)
- ☐ HVAC & catering, kitchen areas (varustelu LVI ja keittiöalueet)
- ☐ Deck outfitting (kansivarustelu)

☐ **Outfitting outside in tents (Lohkovarustelutyö ulkona teltoissa)**

☐ **Outfitting in halls (Lohkovarustelutyö hallessa)**

☐ **Hull production (Runkotuotanto)**
- ☐ Part fabrication (osavalmistus)
- ☐ Block assembly (lohkonkoonti)
- ☐ Grand block assembly (suurlohkokoonti)
- ☐ Hull assembly (rungonkoonti)
- ☐ Surface treatment, (pintakäsittely)

☐ **Other:**____

14. Check the option that best describes your working conditions:

I mainly work alone, without other people around:
Yes ☐ No ☐

I work with other people, in a space that is not too crowded:
Yes ☐ No ☐

I work with other people in a crowded space:
Yes ☐ No ☐

15. Which best describes your working environment:
- ☐ Only indoors
- ☐ Mainly indoors (more than 4 hours/day)
- ☐ Mainly outdoors (more than 4 hours/day)
- ☐ Only outdoors
- ☐ Both equally

16. Average time in hours spent on welding:
- ☐ More than 5 hours/day
- ☐ 3-5 hours/day
- ☐ 1-2 hours/day
- ☐ Not applicable/does not weld

17. Where do you usually have lunch?
- ☐ Canteen Valtameri
- ☐ Canteen Välimeri
- ☐ Near the same place where I work
- ☐ None of the above

18. With whom do you take lunch/breaks (for example, coffee breaks)?
    - ☐ Alone
    - ☐ With colleagues from my company/unit
    - ☐ With colleagues who work elsewhere in the shipyard
    - ☐ None of the above

19. Have you inhaled any of the following agents in your workplace, also considering exposures that may come from the work of other workers?
    Please fill all the lines
    The below information concerns the period of 3 months before start of symptoms.

Exposure type	Never exposed	Exposed less than half your working time	Exposed half or more of your working time	Don't know/not sure
**EXPOSURES INDOORS**				
**Welding fumes**				
**Metal fumes from other sources than welding**				
**Paint or solvent fumes**				
**Metal dust**				
**Wood dust**				
**Sand or stone dust**				
**Fire smoke**				
**Tobacco smoke**				
**Fibre insulation material dust (for example from mineral wool)**				
**Dust from cleaning, brushing, or sweeping**				
**Cleaning chemicals**				
**Other:**				
**EXPOSURES OUTDOORS (INCLUDING TENTS)**				
**Welding fumes**				
**Metal fumes from other sources than welding**				
**Paint or solvent fumes**				
**Metal dust**				
**Wood dust**				
**Sand or stone dust**				
**Fire smoke**				
**Tobacco smoke**				
**Fibre insulation material dust (for example from mineral wool)**				

**Dust from cleaning, brushing, or sweeping**				
**Cleaning chemicals**				
**Other:**				

20. Have you used the following personal protective equipment at your work?
    For each row, please put an “X” in the correct column.
    The below information concerns the period of 3 months before start of symptoms.

**Type of protective equipment**		**Always**	**Most of the time**	**Occasionally**	**Never**	**Not available**
Disposable half mask with particle filters (FFP2 or FFP3)	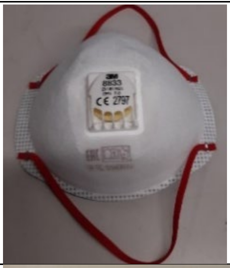					
Half mask with P2-P3 filters, non-powered	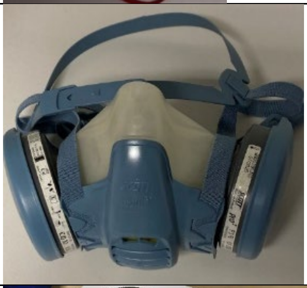					
Powered filtering (P) device incorporating a mask, a helmet, or a hood	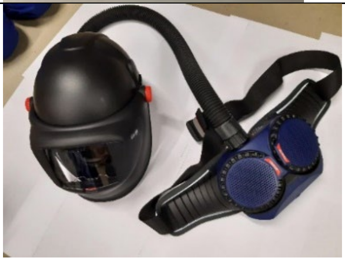					
Continuous flow compressed air line breathing device with welding mask, helmet or hood or mask	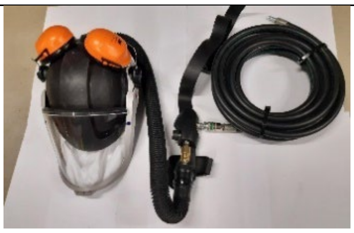					

21. How often have you borrowed/used the same respiratory protective equipment as your workmate?
    - ☐ Every day
    - ☐ 3-4 times a week
    - ☐ Once or twice a week
    - ☐ Only occasionally
    - ☐ Never

Section C: Other risk factors

The below information concerns the period of 3 months before start of symptoms (questions 21-25).

22. How often do you drink alcohol (beer, wine, spirits, etc.)?
    - ☐ Daily or almost daily
    - ☐ 2-3 times a week
    - ☐ 2-4 times a month
    - ☐ Once a month or less
    - ☐ Never

23. Do you smoke?
    - ☐ No, I don’t smoke
    - ☐ I smoked but I stopped
    - ☐ Yes, I smoke

24. If you smoked or you smoke now: on average how many cigarettes per day do you or did you smoke?
    - ☐ Less than 10 cigarettes a day
    - ☐ 10-20 cigarettes a day
    - ☐ More than 20 cigarettes a day

25. Do you use any of the following at the moment?
    - ☐ e-cigarettes / e-vaporizers
    - ☐ nicotine pouches, snuff or chewing tobacco
    - ☐ Other nicotine products (chewing gum, tablets, bandages)
    - ☐ None of the above

26. Do you have any of the following conditions?
    - ☐ Absence of the spleen
    - ☐ Lung disease (such as asthma or COPD)
    - ☐ Kidney disease
    - ☐ Heart disease
    - ☐ Liver disease
    - ☐ Diabetes
    - ☐ I take immunosuppressive medication like corticosteroid tablets, or biological treatment
    - ☐ Immunodeficiency due to illness (such as HIV, leukemia, lymphoma, solid organ transplant, or other)
    - ☐ Other (please specify):

      ____
    - ☐ None

27. How do you travel between home and work (choose the most frequent)?
    - ☐ By public transport
    - ☐ I go alone in my car
    - ☐ I go by car with colleagues
    - ☐ I bike or walk

28. Do you spend time outside the workplace with your co-workers?
    - ☐ Daily or almost daily
    - ☐ 2-3 times a week
    - ☐ 2-4 times a month
    - ☐ Once a month or less
    - ☐ Never

Section D: Vaccination status

Note: 2 previous vaccination campaigns have taken place In Turku shipyard, one in 2019 and one in 2023

29. While working at the shipyard, were you ever offered vaccination against pneumococcal disease by your company/occupational health?
    - ☐ Yes
    - ☐ No
    - ☐ I don't know/not sure

30. Before now, did you get Pneumococcal vaccination (more than one answer is possible)?
    - ☐ yes, in campaign 2019
    - ☐ yes, in campaign 2023
    - ☐ yes, through occupational health while working at the shipyard
    - ☐ yes, in another context
    - ☐ no, never

31. If in question 29 you answered "yes": what type of vaccine was used for the vaccination?
    - ☐ PPV23 (Pneumovax)
    - ☐ PCV20 (Apexxnar)
    - ☐ PCV13 (Prevenar 13)
    - ☐ Other: ___________________________
    - ☐ I don't know/not sure
32. Were you vaccinated against the flu during the winter 2024-2025?
    - ☐ Yes
    - ☐ No
    - ☐ I don't know/not sure

Do you have anything else that you want to tell us?

____
____
____
____

Thank you for your participation!!!

S2: Questionnaire for pneumococcal disease outbreak at the Turku Shipyard in Turku, Finland, 2025

Date of completion (dd/mm/yyyy): _____/_______/________
Questionnaire ID: *Not to be completed* ____________

This questionnaire is part of an outbreak investigation into pneumococcal disease at the Turku Shipyard. The investigation is carried out by the Finnish Institute for Health and Welfare (THL) together with the Wellbeing Services County of Southwest Finland (Varha) with support from the Finnish Institute of Occupational Health (TTL) and the occupational health services of Meyer Turku.

In addition to the shipyard workers who were ill, we invite workers who were not hospitalised with pneumonia to participate in the investigation. Your participation will be useful to understand why the disease was identified among shipyard workers.

Filling out the questionnaire is voluntary. Any information you share with the investigation team will be handled completely confidentially and will not be shared with your employer or any other party. Any information about you will have a number on it instead of your name. In reports of the findings, no individual participants will be identifiable. Any questions regarding the investigation can be addressed to the THL/Varha investigation team on site at the shipyard. By filling in the questionnaire, the participant confirms that consent to participate is given.

Section A: Respondent information

33. Date of birth (dd/mm/year): ___ ___ / ___ ___ / ___ ___ ___ ___
34. Sex: male / female
35. Nationality: ______________________
36. Town or municipality of residence in Finland: ______________________
37. When did you start working at Turku shipyard? (month, year) ______________________________

THIS QUESTIONNAIRE CONCERNS THE PERIOD MARCH – MAY 2025

38. Where do you live when you work?
    - ☐ Hotel/hostel
    - ☐ Apartment/studio
    - ☐ House
    - ☐ Other: _____________________

39. When you work, do you live:
    - ☐ Alone
    - ☐ With work colleagues (how many: ____)
    - ☐ With family members (how many: ____)
    - ☐ With other roommates that do not work at Turku Shipyard (how many: ____)

40. Did you experience any of the following symptoms in the period of March-May 2025?
    - ☐ No symptoms

      OR select all that apply:
    - ☐ Fever (>38 °C)
    - ☐ Shivering/chills
    - ☐ Cough or worsening cough
    - ☐ Shortness of breath
    - ☐ Chest pain or tightness

☐ Other respiratory symptoms: ________________________

41. If you had symptoms, when did the first symptom start? (dd/mm/year) (if you don't remember the exact date, you can give the best estimate): _____/_______/_________

42. Were you hospitalised with pneumonia in the period of March-May 2025?
☐ Yes
☐ No

THIS QUESTIONNAIRE CONCERNS THE PERIOD MARCH – MAY 2025

Section B: Occupational details / work-related questions

43. What is your employer?
☐ Meyer Turku
☐ Contractor (if a contractor, please specify name of the contractor):
____

44. Did you work in any other shipyard within the last year?
☐ Yes (if yes, please specify where and when): ____
☐ No

45. Since you have started working in the shipyard, have you had occupational health checks?
☐ Yes
☐ No

46. Can you describe your main tasks/work in the shipyard?
Please mark all performed activities during the period of March-May 2025:
☐ hitsaaja -welder
☐ levyseppä –plater
☐ operaattori (automatisoidun hitsaus/polttoleikkauskoneen käyttö)- operator (in automated use of welding/oxy-fuel cutting machine)
☐ putkiasentaja- plumber/pipefitter
☐ koneasentaja- mechanic
☐ sähköasentaja- electrician
☐ lvi-asentaja- HVAC-technician/mechanic
☐ sisustusasentaja – interior technician/mechanic
☐ maalari ja ruiskumaalari –(industrial) painters or spray-painters
☐ kunnossapito (sähkö-, nosturi-, koneasentajia)- maintenance and repair (electricians, crane-mechanics, mechanics)
☐ varastotyö- storage worker's work
☐ kuljetusalan työt (trukki, nosturi)- transportation work (crane and forklift-drivers)
☐ siivooja – cleaner
☐ hiekkapuhallus - sandblasting
☐ Any other activities:
____

47. Which sector(s) of the shipyard do you work in (choose all that apply)?
- ☐ **Outfitting ship on wet dock (Varustelutyö laiturissa olevassa laivassa)**
  - ☐ Machinery & Technical decks (konevarustelu)
  - ☐ Interior & Passenger decks (varustelutyö, sisustus ja hyttikannet)
  - ☐ Electrical outfitting (sähkövarustelu)
  - ☐ HVAC & catering, ktichen area (varustelutyö, LVI ja keittiöalueet)
  - ☐ Deck outfitting (kansivarustelu)
- ☐ **Outfitting ship on dry dock (Varustelutyö allasvaiheen laivassa)**
  - ☐ Machinery & Technical decks (konevarustelu)
  - ☐ Interior & Passenger decks (varustelu sisustus ja hyttikannet)
  - ☐ Electricial outfitting (sähkövarustelu)
  - ☐ HVAC & catering, kitchen area (varustelu LVI ja keittiöalueet)
  - ☐ Deck outfitting (kansivarustelu)
- ☐ **Outfitting outside in tents (Lohkovarustelutyö ulkona teltoissa)**
- ☐ **Outfitting in halls (Lohkovarustelutyö halleissa)**
- ☐ **Hull production (Runkotuotanto)**
  - ☐ Part fabrication (osavalmistus)
  - ☐ Block assembly (lohkonkoonti)
  - ☐ Grand block assembly (suurlohkokoonti)
  - ☐ Hull assembly (rungonkoonti)
  - ☐ Surface treatment, (pintakäsittely)
- ☐ **Other:**____

48. Check the option that best describes your working conditions:

    I mainly work alone, without other people around:

    ☐ Yes ☐ No

    I work with other people, in a space that is not too crowded:

    ☐ Yes ☐ No

    I work with other people in a crowded space:

    ☐ Yes ☐ No

49. Which best describes your working environment:
- ☐ Only indoors
- ☐ Mainly indoors (more than 4 hours/day)
- ☐ Mainly outdoors (more than 4 hours/day)
- ☐ Only outdoors
- ☐ Both equally

50. Average time in hours spent on welding:
- ☐ More than 5 hours/day
- ☐ 3-5 hours/day
- ☐ 1-2 hours/day
- ☐ Not applicable/does not weld

51. Where do you usually have lunch (choose the most frequent)?
- ☐ Canteen Valtameri
- ☐ Canteen Välimeri
- ☐ Near the same place where I work
- ☐ None of the above

52. With whom do you take lunch/breaks (for example, coffee breaks)?
- ☐ Alone
- ☐ With colleagues from my company/unit
- ☐ With colleagues who work elsewhere in the shipyard

- [ ] None of the above

53. Have you inhaled any of the following agents in your workplace, also considering exposures that may come from the work of other workers?

Please fill all the lines.

Exposure type (from your work or someone else's work)	Never exposed	Exposed less than half your working time	Exposed half or more of your working time	Don't know/not sure
**EXPOSURE INDOORS**				
Welding fumes				
Metal fumes from other sources than welding				
Paint or solvent fumes				
Metal dust				
Wood dust				
Sand or stone dust				
Fire smoke				
Tobacco smoke				
Fibre insulation material dust (for example from mineral wool)				
Dust from cleaning, brushing, or sweeping				
Cleaning chemicals				
Other:				
**EXPOSURE OUTDOORS (THIS INCLUDES TENTS)**				
Welding fumes				
Metal fumes from other sources than welding				
Paint or solvent fumes				
Metal dust				
Wood dust				
Sand or stone dust				
Fire smoke				
Tobacco smoke				
Fibre insulation material dust (for example from mineral wool)				
Dust from cleaning, brushing, or sweeping				
Cleaning chemicals				
Other:				

54. Have you used the following personal protective equipment at your work?
For each row, please put an "X" in the correct column.

**Type of protective equipment**		**Always**	**Most of the time**	**Occasionally**	**Never**	**Not available**
Disposable half mask with particle filters (FFP2 or FFP3)	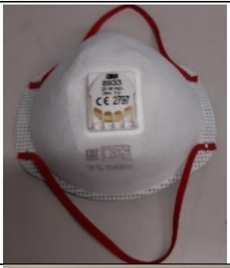					
Half mask with P2-P3 filters, non-powered	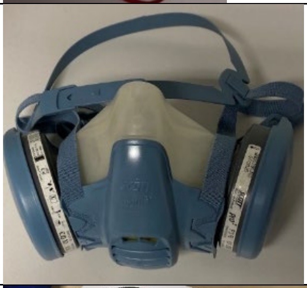					
Powered filtering (P) device incorporating a mask, a helmet, or a hood	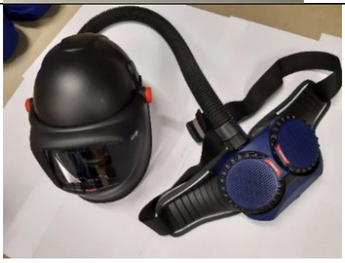					
Continuous flow compressed air line breathing device with welding mask, helmet or hood or mask	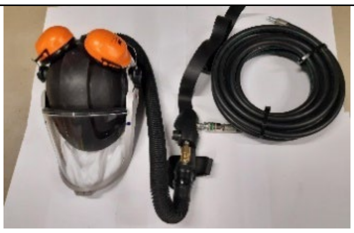					

55. How often have you borrowed/used the same respiratory protective equipment as your workmate?
- ☐ Every day
- ☐ 3-4 times a week
- ☐ Once or twice a week
- ☐ Only occasionally
- ☐ Never

THIS QUESTIONNAIRE CONCERNS THE PERIOD MARCH – MAY 2025

Section C: Other risk factors (tick any that apply)

56. How often do you drink alcohol (beer, wine, spirits, etc.)?
    - ☐ Daily or almost daily
    - ☐ 2-3 times a week
    - ☐ 2-4 times a month
    - ☐ Once a month or less
    - ☐ Never

57. Do you smoke?
    - ☐ No, I don't smoke
    - ☐ I smoked but I stopped
    - ☐ Yes, I smoke

58. If you smoked or you smoke now: on average how many cigarettes per day do you or did you smoke?
    - ☐ Less than 10 cigarettes a day
    - ☐ 10-20 cigarettes a day
    - ☐ More than 20 cigarettes a day

59. Do you use any of the following:
    - ☐ e-cigarettes / e-vaporizers
    - ☐ nicotine pouches, snuff or chewing tobacco
    - ☐ Other nicotine products (chewing gum, tablets, bandages)
    - ☐ None of the above

60. Do you have any of the following conditions?
    - ☐ Absence of the spleen
    - ☐ Lung disease (such as asthma or COPD)
    - ☐ Kidney disease
    - ☐ Heart disease
    - ☐ Liver disease
    - ☐ Diabetes
    - ☐ I take immunosuppressive medication like corticosteroid tablets, or biological treatment
    - ☐ Immunodeficiency due to illness (such as HIV, leukaemia, lymphoma, solid organ transplant)
    - ☐ Other (please specify):

      ____
    - ☐ None

61. How do you travel between home and work (choose the most frequent)?
    - ☐ By public transport
    - ☐ I go alone in my car
    - ☐ I go by car with colleagues
    - ☐ I bike or walk

62. Do you spend time outside the workplace with your co-workers?
    - ☐ Daily or almost daily
    - ☐ 2-3 times a week
    - ☐ 2-4 times a month
    - ☐ once a month or less
    - ☐ Never

Section D: Vaccination status

Note: 2 vaccination campaigns have taken place in Turku shipyard, one in 2019 and one in 2023.

63. While working at the shipyard, were you ever offered vaccination against pneumococcal disease by your company/occupational health?
    - ☐ Yes
    - ☐ No
    - ☐ I don't know/not sure

64. Before now, did you get Pneumococcal vaccination (more than one answer is possible)?
    - ☐ yes, in campaign 2019
    - ☐ yes, in campaign 2023
    - ☐ yes, in campaign 2025
    - ☐ yes, through occupational health while working at the shipyard
    - ☐ yes, in another context
    - ☐ no, never
    - ☐ I don't know/not sure
65. If in question 32 you answered "yes": what type of vaccine was used for the vaccination (more than one answer is possible)?
    - ☐ PPV23 (Pneumovax)
    - ☐ PCV20 (Apexxnar)
    - ☐ PCV13 (Prevenar 13)
    - ☐ Other: ___________________________
    - ☐ I don't know/not sure

66. Were you vaccinated against the flu during the winter 2024-2025?
    - ☐ Yes
    - ☐ No
    - ☐ I don't know/not sure

Do you have anything else that you want to tell us?

____

____

____

____

Thank you for your participation!!!
